# Comparison of the endocranial- and brain volumes in brachycephalic dogs, mesaticephalic dogs and Cavalier King Charles spaniels in relation to their body weight

**DOI:** 10.1186/1751-0147-56-30

**Published:** 2014-05-13

**Authors:** Martin J Schmidt, Kerstin H Amort, Klaus Failing, Melanie Klingler, Martin Kramer, Nele Ondreka

**Affiliations:** 1 Department of Veterinary Clinical Sciences Small Animal Clinic, Justus Liebig-University, Frankfurter Strasse 108, D-35392 Giessen, Germany; 2 Unit for Biomathematics and Data Processing, Faculty of Veterinary Medicine Justus Liebig-University, Frankfurter Strasse 108, D-35392 Giessen, Germany

**Keywords:** Chiari malformation, Syringomyelia, Allometry, Brachycephaly, Cavalier King Charles spaniel

## Abstract

**Background:**

A number of studies have attempted to quantify the relative volumes of the endocranial volume and brain parenchyma in association with the pathogenesis of the Chiari-like malformation (CLM) in the Cavalier King Charles spaniel (CKCS). In our study we examine the influence of allometric scaling of the brain and cranial cavity volume on morphological parameters in different dog breeds. MRI scans of 110 dogs (35 mesaticephalic dogs, 35 brachycephalic dogs, 20 CKCSs with SM, and 20 CKCSs without SM) have been used to create 3-dimensional volumetric models of skull and brain parts. Volumes were related to body weight calculating the adjusted means for different breeds.

**Results:**

There was a strong global dependency of all volumes to body weight (*P* < 0.0001). The adjusted means of the absolute and relative volumes of brain parenchyma and cranial compartments are not significantly larger in CKCSs in comparison to brachycephalic and mesaticephalic dogs. A difference in absolute or relative volumes between CKCSs with and without SM after relating these values to body weight could not be identified. The relative volume of the hindbrain parenchyma (caudal fossa parenchyma percentage) was larger in brachycephalic dogs than in CKCSs, without causing herniation or SM.

**Conclusion:**

An influence of body weight exist in dogs, which can be sufficiently large to render conclusions on the difference in volumes of the brain and skull unsafe unless some account of the body weight is taken in the analysis. The results of this study challenge the role of overcrowding for the development of SM in dogs.

## Background

A number of studies have been conducted that determined the relative volumes of brain parts and endocranial compartments to explain the pathogenesis of the Chiari-like malformation (CLM) in the Cavalier King Charles spaniel (CKCS) [1-8]. CLM is characterized by foramen magnum cerebellar herniation, which was suggested to be a consequence of disproportionate growth between the cranial cavity volume and hindbrain parenchyma [9]. Mesaticephalic dogs (MCs), brachycephalic dogs (BCs) and CKCSs have been compared and aberrant dimensions of brain and skull volumes have been found in the CKCS. Initial studies suggested a smaller caudal cranial fossa and an overcrowding of this compartment in CKCSs in general or at least in CKCSs with SM [1,4]. Subsequent studies failed to find abnormalities of this skull compartment [5-7]. It was also suggested that CKCSs have a proportionally larger hindbrain in relation to a normal caudal cranial fossa, expressed by the so called caudal fossa parenchyma percentage (CFPP) [2,4,7]. Recently, a larger cerebellar volume in relation to the cerebral volume was determined [8].

It is important to know that such volume calculations of brain and skull can be influenced by the body weight of the examined animals. Changes in brain mass in mammals occur in individual proportion to the increase of body mass of the animals (allometric scaling, [10-12]). With increasing body size the increased muscle mass and increase of peripheral receptors also lead to an increase of the corresponding central representation fields in the cerebral cortex (motor cortex, somato-sensory cortex). This causes an increase of brain mass in a larger individual of a species in a determined proportion to the increase of body weight [10-12]. In most previous studies, dogs of various weight groups ranging from 1 to 40 kg have been compared [2,6-8]. Differences between brain-skull relations found in these dogs might simply reflect the general variance between larger and smaller animals [10-12]. In addition, it is also expected that all dog breeds share the same proportions between brain and braincase and the same allometric increase of these proportions. It must be considered that larger animals have more space in their skull that is not occupied by the brain, than smaller animals [10,13,14]. Furthermore, general differences of the proportions between brain volume and endocranial volume in dogs with brachycephalic and mesaticephalic head morphology have also not been taken into account.

We hypothesize that all brain and skull volumes are strongly influenced by body size and the relations between brain and skull dimensions differ between small and large dogs in general. We therefore calculated volumes and relative proportions of the brain and skull in relation to the body weight of dogs. The results of this study will be discussed with special reference to the role of caudal cranial fossa overcrowding in the pathogenesis of CLM and SM in the CKCS. We also hypothesize that overcrowding is not a prerequisite for CLM and SM as it has been shown in human patients with Chiari malformation [15-17].

## Methods

### Animals

The archive of magnetic resonance imaging (MRI) scans of the Justus Liebig University (JLU) was retrospectively searched for cranial studies of dogs. MRI scans of 110 dogs were chosen to create 4 groups. Group 1 included 35 MCs, group 2 included 35 BCs. All dogs had been examined for epilepsy diagnosis. Only dogs ≥ 5 years of age without evidence of central nervous system lesions were included. Dogs with cerebellar displacement into the foramen magnum or SM were excluded. In addition, MRI-data of 40 CKCSs were chosen from the archive, all of which underwent MRI-scanning of the head and cervical spine for breeding selection. All these CKCSs had CLM, which defined as evidence of caudal cerebellar herniation into the foramen magnum or indentation by the supraoccipital bone, irrespective of the presence of SM [18]. 20 CKCSs with SM (group 3) and 20 CKCSs without SM (group 4) were chosen. SM was defined as a fluid-containing cavity within the spinal cord parenchyma with a transverse diameter of greater than or equal to 2 mm [6]. Only CKCS ≥ 5 years were included as SM can be a late onset disease [6]. Dogs in group 3 and 4 were thoroughly weight matched. In both groups there were 10 dogs ≤ 8 kg and 10 dogs > 8 kg.

### MR image analysis

**Figure 1:**
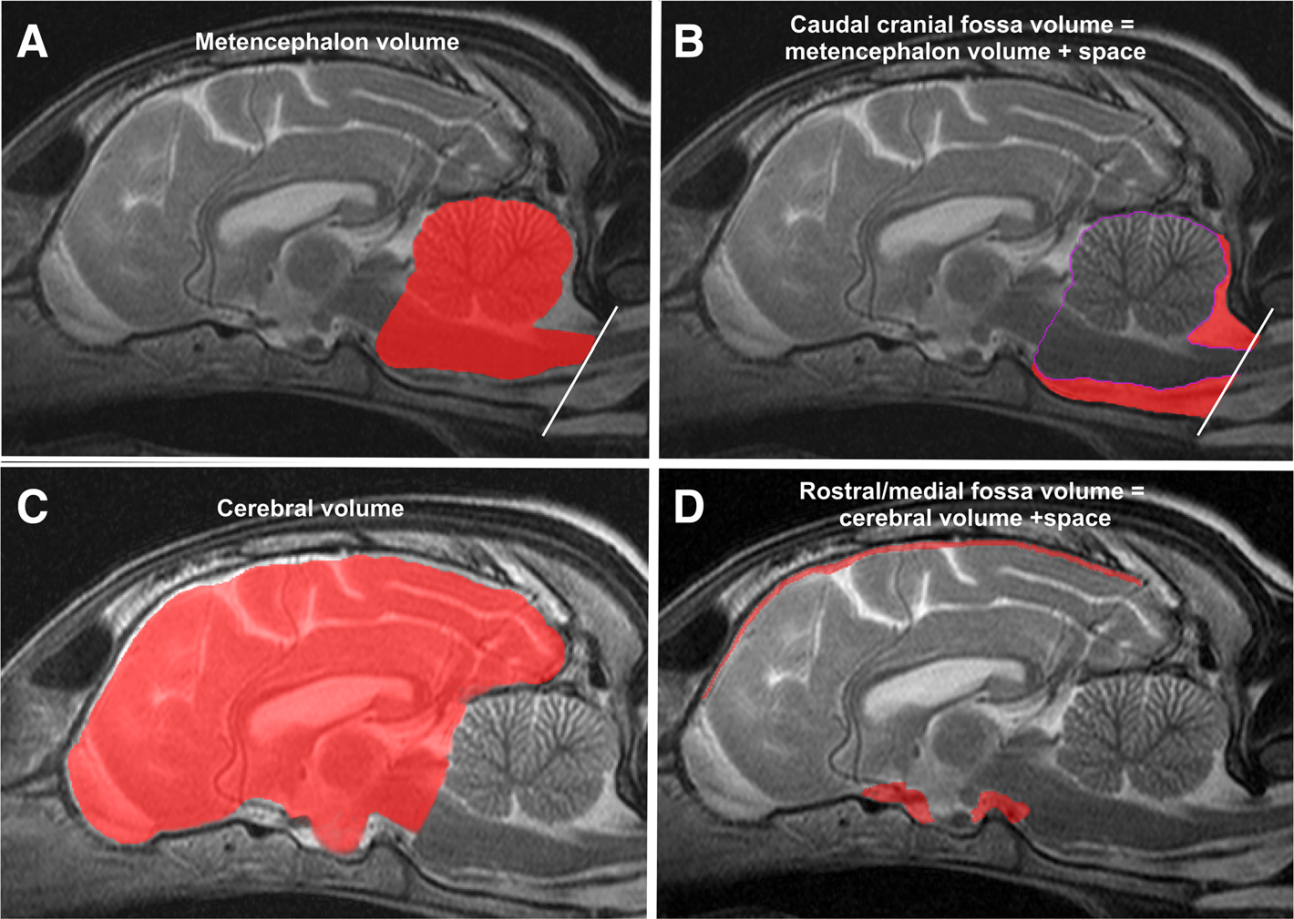
**Image segmentation for volume determination.** Image segmentation of cranial compartments and their parenchymal contents. The delineation of the metencephalic volume and the cerebral volume (or caudal cranial fossa volume and rostral and middle cranial fossa volume respectively) was set along the contour of the rostral aspect of the cerebellum and a line connecting the touching point of the cerebellum with the brainstem to the rostral border of the pons **(A-C)**. The caudal boundary of the caudal cranial fossa volume and the metencephalic volume was a line between the intercondylar incisures and the most caudal point of the foramen magnum (**A**, **B**, white line). The caudal cranial fossa volume was measured by adding the volume of the cerebellum and brainstem (**A**: marked in red) to the CSF subarachnoid space that surrounds it (**B**: marked in red) in this defined compartment. The rostral and middle cranial fossa volume and cerebral volume were measured in the same manner using the first contour as a caudal end **(C, D)**. The total brain volume was calculated as the sum of metencephalic volume and cerebral volume, the total endocranial volume was calculated as the sum of the total brain volume and subarachnoid space.

The volumes of the endocranial volume and the brain were determined based on MRI datasets using a 1.0 Tesla scanner (Gyroscan Intera, Phillips, Hamburg, Germany). Transverse and sagittal T2-weighted images of the head (T2-Turbospin echo, TE 120 ms, TR: 2900 ms, slice thickness 2mm) were chosen for image segmentation (slice thickness 2.5 mm, gap: 0.5 mm). Field of view was 180 × 180 mm in small dogs and 210 × 210 mm in large dogs. Matrix was 288 × 288 in small dogs and 384 × 384 in large dogs leading to a pixel size between 0.625 × 0.625 mm and 0.54 × 0.54 mm. Image processing for volume rendering was achieved using graphical software (AMIRA®, Mercury Computers Systems, Berlin, Germany). This program combines image information of two or more different planes which allows accurate manual image segmentation on a slice-by-slice basis. Segmentation techniques were previously described in detail [5]. MRI-based volume measurements of the brain-parts are routinely performed in veterinary medicine. The accuracy of the technique even for small volumes has been proven in veterinary neuroradiology [19,20]. The observer of the images was blinded to breed, age and the presence of SM. Masks were created in transverse and sagittal planes from individual slices by free-hand measurements. All brain volumes included the volume of the ventricular system. Volumes of interest were: The total brain volume, the metencephalon volume (cerebellum and caudal brainstem) and the cerebral volume, the total endocranial volume, the caudal cranial fossa and the rostral and middle cranial fossa volume and the space in the cranial cavity that was not occupied by parenchyma. The delineation of metencephalic and cerebral volume (or caudal cranial fossa volume and the rostral and middle cranial fossa volume respectively) was set along the contour of the rostral aspect of the cerebellum and a line connecting the touching point of the cerebellum with the brainstem to the rostral border of the pons (Figure 1A-C). The caudal boundary of the caudal cranial fossa volume and the metencephalic volume was a line between the intercondylar incisures and the most caudal point of the foramen magnum (Figure 1A, B). The caudal cranial fossa volume was then measured by adding the volume of the cerebellum and brainstem (marked in red in Figure 1A) to the subarachnoid space (marked in red in Figure 1B) that surrounds it in this defined compartment. The rostral and middle cranial fossa volume and cerebral volume were measured in the same manner using the first line as a caudal end (Figure 1C, D). The total brain volume was calculated as the sum of metencephalic volume and cerebral volume. The total endocranial volume was calculated as the sum of the total brain volume and subarachnoid space. The CFPP was defined as the quotient of the metencephalic volume and the caudal cranial fossa volume.

### Statistical analysis

Statistical analysis was performed using a commercial software package (Graph Pad Prism 4.0, Graph Pad Software Inc., and San Diego, California). The deviation from normal distribution was checked using the normal probability plot of model residuals for each variable. To evaluate the relationship between the measured volumes and body weight and to compare the adjusted means between all groups a one-way analyses of covariance (ANCOVA) was performed for each parameter. If the influence of the covariable body weight (global regression coefficient) was statistically significant the calculated means of the group parameters were corrected for body weight (adjusted means). The adjusted means are sample means adjusted to a mean body weight and regression coefficient within a group [21]. In a second step the adjusted means are checked for significant global differences between groups. If significant statistical differences were given the groups were compared pairwise using the *t*-test with Bonferroni-Holm correction. Results of this calculation provide a comparison of the variables between groups corresponding to the same bodyweight given as geometric mean at 10 kg in the interbreed comparison.

Within the analysis of covariance the global dependency of the variables on the logarithm of the body weight was tested in a second step. Furthermore the slopes of the regression lines were compared. The slope for the CFPP is calculated per kg body weight.

After the interbreed comparison (MCs *vs.* BCs dogs *vs.* CKCSs) the same calculations were made for intrabreed comparison (CKCSs with and without SM). Adjusted means refer to 8.36 kg in the comparison of the latter groups.

In a last step the values between the CKCSs of body weight ≤ 8 and > 8 kg within group 3 and 4 were analyzed using a student’s *t*-test. Fischer`s exact tests were used to test for differences of the proportion between male and female dogs in all groups. For all comparisons a family-wise significance level of P < 0.05 was used.

## Results

### Animals

Group 1 (MCs) comprised 20 male and 15 female dog weighing 2–49 kg. Group 2 included 13 male and 22 female dogs weighing 1.8 to 25 kg. Breeds and numbers of dogs in the groups are listed in Tables 1 and 2. In group 3 (CKCSs with SM) 8 dogs were male and 12 female. Age of the CKCSs was not normally distributed. Their age range was 5–8 years (median: 5.63), their weight ranged from 4 to 14.5 kg. Group 4 (CKCSs without SM) included 9 male and 11 female dogs. Their weight ranged from 4 to 17 kg, age range was 5–9 years (median: 5.76).

**Table 1:** Number of dogs of each breed included in the mesaticephalic dog group

**Mesaticephalic dog breeds**	**Body weight**
West Highland White terrier (n = 2)	6-7 kg
Münsterländer dog (n = 2)	22 kg; 25 kg
Schnauzer (n = 3)	14.5-18.5 kg
Alaskan Malamute (n = 3)	36-42 kg
Hovawart (n = 2)	44 kg; 46 kg
Weimaraner (n = 3)	35-42 kg
German Pinscher (n = 1)	14 kg
Poodle (n = 4)	29-32 kg
Jack Russel terrier (n = 3)	5 - 6.5 kg
Fox terrier (n = 2)	8.5 kg; 9 kg
Pommeranian (n = 1)	2.1 kg
Australian Shepherd (n = 4)	25-30 kg
Airedale terrier (n = 1)	20 kg
Dachshund (n = 1)	4.5 kg
Bernese Mountain dog (n = 2)	42 kg; 45 kg
St Bernards (n = 1)	49 kg

**Table 2:** Number of dogs of each breed included in the brachycephalic dog group

**Brachycephalic dog breeds**	**Body weight**
Pug (n = 5)	6.2-9 kg
Boston terrier (n = 4)	9 -14 kg
English bulldog (n = 3)	15-25 kg
French bulldog (n = 4)	9-12 kg
Pekingese (n = 3)	3-5 kg
Maltese (n = 3)	3-4.8 kg
Bolonka Zwetna (n = 2)	2.2kg; 3 kg
Papillon (n = 2)	2.5 kg; 2.8 kg
Yorkshire terrier (n = 4)	1.8-2.9 kg
Shih Tzu (n = 3)	7.5-8.2 kg
Chihuahua (n = 2)	2.4 kg; 3 kg

There was no significant difference of the proportion between male and female dogs (MCs *vs.* BCs, *P* = 0.1559; MC *vs.* all CKCSs, *P* = 0.2506; BCs *vs.* all CKSSs, *P* = 0.8155, CKCSs *vs.* CKCSs, *P* = 1). The age of the CKCSs in group 3 was not significantly different from group 4 (*P* = 0.32).

For all parameters a good approximation of normal distribution was found in the residuals of the ANCOVA-model. Interbreed comparison of the adjusted means and the slopes of the regression lines are summarized in Table 3. All volumes show a high global dependency on bodyweight (*P* < 0.001). BCs and CKCSs have a significantly larger total brain volume at adjusted body weight than MCs (*P* = 0.0011), which is equally distributed to the cerebral volume and metencephalic volume. Total brain volume is not different between BCs and CKCSs. The slope of total brain volume is the significantly steeper in BCs (*P* = 0.009) and MCs also have a significantly steeper slope of total brain volume then CKCSs (*P* = 0.026). Both, infratentorial and supratentorial parenchyma increase with body weight. MCs have a lower adjusted means (*P* = 0.0015) and a lower slope of metencephalic volume (*P* = 0.015).

**Table 3:** **Results of the one-way analysis of covariance and pairwise t-tests of *inter*** **breed comparison**

**Variable**	**Group**	**Adjusted means (at body weight = 10 kg)**	**Equality of the adjusted means (p-value)**	**Global regression coefficient**		**Equalitiy of slopes**	
				**Estimate**	***P*-value**	**Estimates**	***P*-value**
**Total brain volume**	CKCS:	81.07 cm^3^ ± 1.03	**0.0011**	47.13 ± 2.96	**< 0.001**	35.31 ± 7.67	**0.009**
	brachycephalic:	84.30 cm^3^ ± 1.41					
	mesaticephalic:	**77.18** cm^3^ ± 1.41					
						**56.75** ± 4.82	
						**42.39** ± 4.44	**0.026**
**Cerebral volume**	CKCS:	70.98 cm^3^ ± 1.15	**0.041**	39.18 ± 2.83	**< 0.001**	27.75 ± 7.99	0.23
	brachycephalic:	69.83 cm^3^ ± 1.51					
	mesaticephalic:	**65.70** cm^3^ ± 1.57				43.80 ± 4.77	
						38.21 ± 4.00	
**Metencephalic volume**	CKCS:	11.6 cm^3^ ± 0.11	**0.0015**	6.34 ± 0.40	**< 0.001**	7.57 ± 0.87	**0.015**
	brachycephalic:	11.79 cm^3^ ± 0.2					
	mesaticephalic:	**10.91** cm^3^ ± 0.20				7.21 ± 0.68	
						**5.39** ± 0.64	
**Total endocranial volume**	CKCS:	83.93 cm^3^ ± 7.13	**0.0015**	52.55 ± 3.10	**< 0.001**	35.04 ± 8.14	
	brachycephalic:	**85.83 cm**^**3**^ ± 7.67					
	mesaticephalic:	81.53 cm^3^ ± 8.65				**61.47** ± 4.79	**0.024**
						**49.65** ± 4.76	**0.009**
**Rostral and middle cranial fossa volume**	CKCS:	69.84 cm^3^ ± 1.31	0.21	41.06 ± 3.17	**< 0.001**	32.46 ± 8.51	0.15
	brachycephalic:	72.94 cm^3^ ± 1.66					
	mesaticephalic:	70.08 cm^3^ ± 1.71				43.52 ± 5.98	
						43.02 ± 4.22	
**Caudal cranial fossa volume**	CKCS:	12.51 cm^3^ ± 0.21	0.28	8.01 ± 0.53	**< 0.001**	8.38 ± 1.18	0.76
	brachycephalic:	12.90 cm^3^ ± 0.28					
	mesaticephalic:	12.20 cm^3^ ± 0.29				8.45 ± 1.10	
						7.63 ± 0.78	
**Subarachnoid space**	CKCS:	4.67 cm^3^ ± 0.08	0.63	6.09 ± 0.41	**< 0.001**	**6.52** ± 0.67	**0.009**
	brachycephalic:	4.54 cm^3^ ± 0.11					
	mesaticephalic:	4.44 cm^3^ ± 0.29				4.73 ± 0.38	
						**7.05** ± 0.92	**0.023**
**Caudal fossa parenchyma percentage**^**§**^	CKCS:	0.89 ± 0.001	**< 0.0001**	-0.00287 ± 0.00021	**< 0.001**	-0.0046 ± 0.00048	
	brachycephalic:	**0.91** ± 0.003					
	mesaticephalic:	0.86 ± 0.004					
						**-0.0056 **± 0.00058	**< 0.001**
						**-0.0024** ± 0.00027	**< 0.001**

^§^For the analysis of CFPP body weight was **not** transformed by logarithm.

The table presents results of the comparison of the equality of regression coefficients (slopes) on the logarithm of the body weight and the equality of adjusted means at BW = 10 kg between the different groups. Significantly different estimates and p-values are bold-typed and represent the result of the pairwise comparison. Normal typed estimates and p-values are not significantly different and represent the result of the global comparison.

BCs have a significantly higher adjusted means of the total endocranial volume (*P* = 0.0015) and the steepest slope (*P* = 0.024). CKCSs and MCs are not significantly different. The slope of the endocranial volume is significantly steeper in MCs than in CKCSs (*P* = 0.009), and BCs had a significantly steeper slope than MCs (*P* = 0.024). In CKCSs and BCs a significantly lower increase of space with increasing body weight compared to MCs was found (*P* = 0.023). Furthermore, the increase was significantly less in BCs dogs compared to CKCSs (*P* = 0.009).

The adjusted means of the CFPP was significantly largest in BCs (*P* <0.0001). The slope of the CFPP of the MCs decreases significantly less (*P* <0.001) and in BCs significantly more than CKCSs (*P* <0.001).

Results of the intrabreed comparison of CKCS with and without SM are summarized in Table 4. There was no significant difference in volumes between CKCSs with and without SM.

**Table 4:** **Results of the one-way analysis of covariance and pairwise t-tests of the *intra*** **breed comparison**

**Variable**	**Group**	**Adjusted means (at BW = 8.36)**	**Equality of the adjusted means ( *P *-value)**	**Global regression coefficient**		**Equalitiy of regression coefficients**	
				**Estimate**	***P*-value**	**Estimates**	***P*-value**
**Total brain volume**	SM	79.02 ± 1.45	0.48	34.02 ± 7.93	**< 0.001**	44.28 ± 11.37	0.087
	No SM	77.50 ± 1.55				16.05 ± 9.60	
**Cerebral volume**	SM	68.61 ± 1.50	0.42	26.19 ± 8.24	**0.003**	35.65 ± 11.68	0.130
	No SM	66.78 ± 1.62				9.62 ± 10.61	
**Metencephalic volume**	SM	10.99 ± 0.16	0.90	7.59 ± 0.90	**< 0.001**	7.99 ± 1.33	0.57
	No SM	11.02 ± 0.18				6.90 ± 1.16	
**Total endocranial volume**	SM	83.02 ± 1.53	0.44	33.54 ± 8.40	**< 0.001**	40.45 ± 12.53	0.28
	No SM	81.25 ± 1.56				21.44 ± 9.91	
**Rostral and middle cranial fossa volume**	SM	69.23 ± 1.62	0.95	23.33 ± 8.95	**0.013**	29.55 ± 13.15	0.37
	No SM	69.09 ± 1.71				12.77 ± 11.47	
**Caudal cranial fossa volume**	SM	11.98 ± 0.17	0.074	9.82 ± 0.96	**< 0.001**	9.92 ± 1.42	0.89
	No SM	12.53 ± 0.19				9.64 ± 1.22	
**Subarachnoid space**	SM	4.31 ± 0.12	0.090	6.26 ± 0.67	**< 0.001**	6.87 ± 0.94	0.23
	No SM	3.99 ± 0.13				5.18 ± 0.92	
**Caudal fossa parenchyma percentage**^**§**^	SM	0.89 ± 0.002	0.73	-0.0045 ± 0.00051	**< 0.001**	-0.0058 ± 0.00052	0.21
	No SM	0.89 ± 0.002				-0.0064 ± 0.00098	

^§^For the analysis of CFPP body weight was **not** transformed by logarithm.

The table presents the results of the comparison for Cavalier King Charles spaniels (CKCS) to test the equality of the regression coefficients (slopes) on the logarithm of the body weight (BW) and the equality of adjusted means at BW = 8.36 kg between the CKCSs with and without SM (SM). Significantly different estimates and p-values are bold-typed and represent the result of the pairwise comparison. Normal typed estimates and p-values are not significantly different and represent the result of the global comparison.

**Figure 2:**
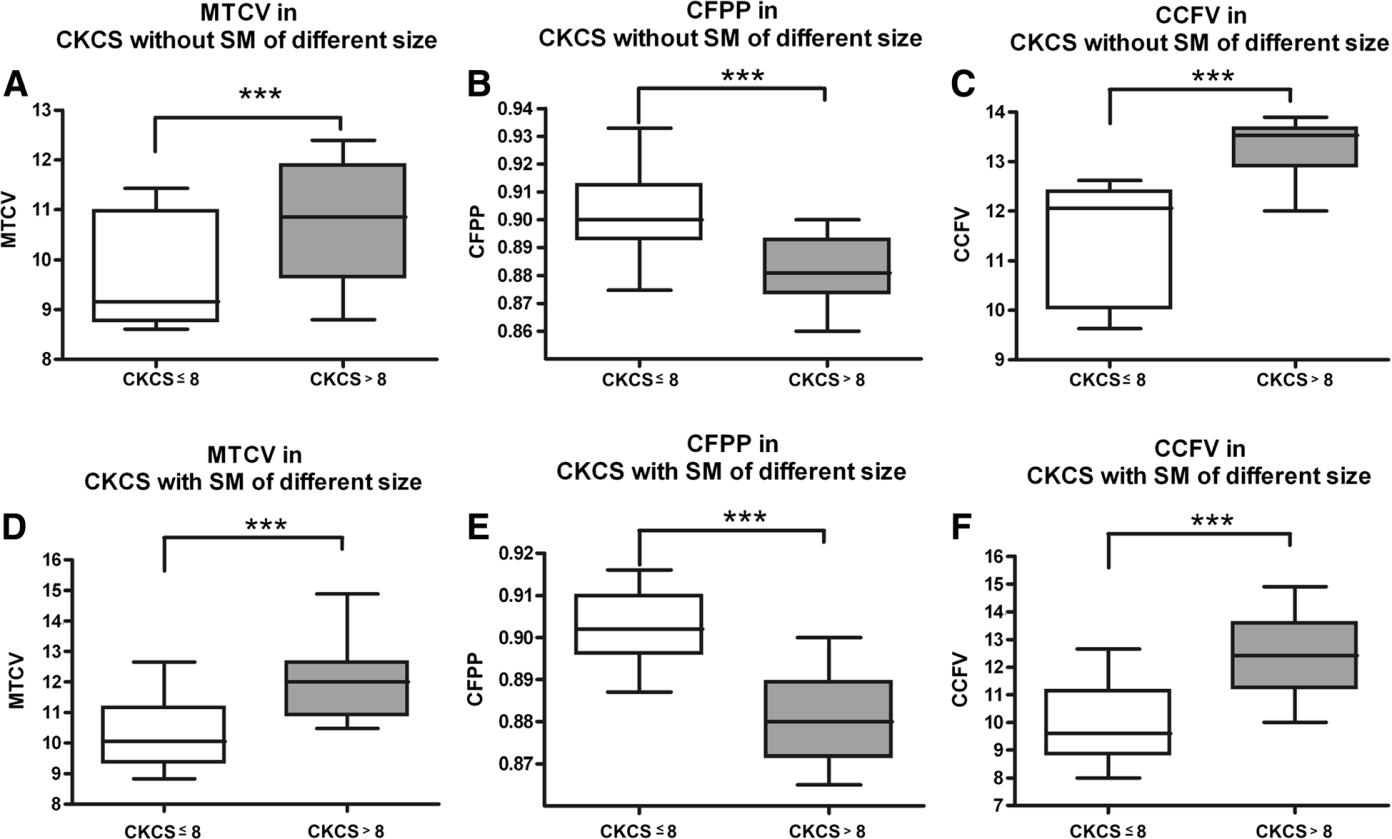
**Results of the group comparison of Cavalier King Charles spaniels ≤ 8 and ≥ 8 kg body weight.** The box-and whisker plots show the comparison of calculated parameters of CKCS ≤ 8 and ≥ 8 kg body weight. The metencephalic volume (MTCV), the caudal fossa parenchyma percentage (CFPP) and the caudal cranial fossa volume (CCFV) in Cavalier King Charles Spaniels with a body weight ≤ 8 and ≥ 8 kg with SM **(A-C)** and without SM **(D-F)** are compared. Results of the ANOVA presented as median, range, 25, and 75 quartile in a box and whisker plot. All parameters are significantly different between dogs ≤ 8 kg and > 8 kg in both groups, clearly presenting the influence of body weight.

Median weight of dogs ≤ 8 in group 3 was 4.85 kg and 4.74 kg in group 4, median weight of dogs > 8 kg in group 3 was 10.9 kg and 10.5 kg in group 4. Medians of the body weights were not significantly different (*P* = 0.9 and *P* = 0.58 respectively). All brain and skull volumes are significantly different between the dogs ≤ 8 and > 8 kg irrespective of the presence of SM (*P* <0.0001). Figure 2 exemplary shows the comparison of metencephalic volume, CFPP and caudal cranial fossa volume CKCSs ≤ 8 and > 8 kg in dogs with and without SM.

## Discussion

The present study compares volumes of the brain and cranial cavity in different dog breeds in relation to their body weight using the adjusted means. The results demonstrate a strong correlation between brain and skull volumes and body weight in the dogs of our study. This is in agreement with other studies that have determined body size-brain volume relations in mammals in general [10,12,22] and in canids in particular [1,14,23-25]. Our approach follows the assumption of a basic uniformity of the brain in all dog breeds. We do not consider other non-allometric factors based on selection related to behavioral and environmental factors affecting brain size independent from selection for body size by breeders. Some breeds have been selectively bred for the “quality of learning” which has been upstaged in other breeds in favor of other characteristics. MCs have been reported to have a higher trainability than brachycephalic dogs which possibly could have an influence on the number and connectivity of neurons [26]. This could have an influence on the calculated relations if these influences would be large enough to outmatch the influence of bodyweight. We also not consider the influence of obesity. The fat free body weight would have been the ideal parameter to show the precise association between body weight and brain mass in dogs. However, the determination of a definite scaling exponent for the domestic canine species was not the aim of this study. We simply wanted to emphasize the general dependency of the calculated brain and skull parameters on the variable body weight and its implications for the results of previous studies.

### Comparison of brain volumes

We found that BCs and CKCSs have a larger total brain volume relative to body weight than MCs. Such intraspecific variability of relative brain size has been previously demonstrated in domestic animals, especially when dwarfs have evolved within a species [10,12,22,25]. The restriction of “postcranial growth” in small dog breeds is of importance since the postnatal increase in brain mass finishes around six month and completion of skeletal growth in dogs occurs later in postnatal development [27-29]. It has been shown, that skeletal growth is diminished in BCs due to hormonal deficits or epiphyseal dysfunction of long bones and spine consistent with achondroplasia or ateliotic dwarfism, whereas brain growth is less influenced in these breeds [30-33]. This impaired skeletal growth accounts for the proportionally highest total brain volume compared to body weight in BCs. A selectively larger hindbrain in CKCSs in comparison to other BCs could not be determined after correction for body weight.

Our calculations show that MCs have a significant lower adjusted means and lower slope of the metencephalic volume. The reason for this finding in the heavier MCs is that cerebral white matter increases disproportionately in larger brains due to an increase of neocortical interconnections [10,34]. The white matter of cerebellum lacks comparable cortico-cortical connections [35]. In contrast to the white matter of the cerebrum, cerebellar white matter does therefore not hyperscale relative to gray matter [34]. The increase of the total brain volume is mainly caused by the forebrain in larger animals. The hindbrain lags behind the allometric increase of the forebrain in most mammals [36].

This finding has important implications for studies aiming the comparison of relative brain volumes of CKCSs with MCs [2,4,8], because large MCs have lower hindbrain volumes than small MCs. Labradors, which can weigh up to 40 kg, have been commonly used as a control group for CKCSs [2,4,8]. However, it must be considered that this large breed can have a physiological small CFPP.

### Comparison of endocranial volumes

We demonstrated that the BCs of our study have the highest adjusted means of the total endocranial volume and the highest slope. However, as the total endocranial volume is a value that is strongly correlated to the total brain volume, the dog breeds with the highest total brain volume most likely have the correlative highest total endocranial volume in this statistical calculation. The comparison of the subarachnoid space is less influenced by the total brain volume and better reflects the differences in skull growth between groups. In CKCSs and BCs a lower increase of subarachnoid space with increasing body weight compared to MCs was found. Furthermore, the increase was significantly less in BCs compared to CKCSs. Generally, the volume of the cranial cavity exerts the volume of the whole brain in inter- and intraspecies comparisons [10,14,37]. The skull growth is not completed until 12 month post partum in dogs [13,38,39]. In brachycephalic animals the growth of the synchondroses is thought to be impaired resulting in reduced longitudinal extension of the cranial base as well as in the long bones [31,32]. Although the CKCS have been identified as an extreme BC [40] a smaller adjusted means of the subarachnoid space in comparison to other BCs could not be determined. In fact, the latter group show less increase in subarachnoid space with increasing body weight than CKCSs. The rostral and middle cranial fossa and the caudal cranial fossa are not significantly different between groups. This can be seen as further evidence against the former theory that through the minituarisation process in dogs both the brain and skull are proportionally smaller, but in the CKCS only the cranium is reduced in volume [4].

### Relative skull-brain dimensions

We could not confirm that CKCSs have a larger CFPP in comparison to BCs or MCs. In fact, the adjusted means of the CFPP was largest in BCs. Although BCs have a higher CFPP, this has not caused cerebellar herniation or SM in one of the dogs of this study. Therefore, the concept of increased crowding of the caudal cranial fossa cannot be regarded as the major pathogenetic factor for cerebellar herniation or the development of SM in the CKCS, because crowding is even more severe in other BCs. The slope of the CFPP in MCs has been found to decrease significantly less in proportion to increased body weight. This should be interpreted as a statistical artifact, because if BCs have more total brain volume and less intracranial space with increased body weight, they should have the slowest slope of caudal fossa parenchyma percentage. The higher upper range of the body weight in MCs causes the deviation of the regression line to the right creating a flatter slope, which would not be observed if the upper weight range of the MCs would be the same as in the BCs and CKCSs.

Our results concerning these differential growth tendencies have important implications for comparative studies of brain and skull dimensions in dogs. A comparison of the CFPP between BCs and CKCSs with MCs as a control group as must be assessed with highest caution. As we have shown, the volume of brain parenchyma increases with increasing body weight in MCs as well as BCs and CKCSs but the cranial cavity volume does not increase in the same amount in the BCs and CKCSs. These growth differences can again give room for errors when trying to calculate relative dimensions between brain and skull volumes. These relations change if either the brain volume increases or the cranial cavity volume decreases. In the CKCS we can find both scenarios with increased body weight. Large CKCSs can have larger absolute brain volumes than other BCs and their endocranial volume does not increase as much as in large MCs. The combination of these features can explain findings of a similar CFPP in CKCSs and Labrador retrievers [4] that must by no means be caused by an abnormally large hindbrain in the CKCS. In addition, as mentioned above, the tendency to have less metencephalic volume in larger MCs further influences this relation.

The comparison of adjusted brain or skull volumes between CKCSs with and without SM could not reveal the same differences as in previous studies without adjustment for body weight. We could rather find significant differences between CKCS ≤ 8 and > 8 kg in CKCS with and without SM. This clearly presents a possible bias in studies using non-weight matched groups on calculations of brain and skull dimensions in dogs.

## Conclusion

It is important to realize that an influence of body weight exist in dogs, which can be sufficiently large to render conclusions on the difference in volumes of the brain and skull unsafe unless some account of the body weight is taken in the analysis. Future studies comparing volumes of brain parts and skull compartments in dogs and especially in the CKCS should use thoroughly weight matched groups. Also considering possible influence of other non-allometric factors, control groups should only comprise dog breeds whose physiognomy, growth features, and selection pressure on cognitive abilities is comparable to the CKCS.

Based on our results we challenge the importance of overcrowding for the development of CLM and SM in CKCSs.

## Abbreviations

BC: Brachycephalic dog; BW: Body weight; CCFV: Caudal cranial fossa volume; CLM: Chiari-like malformation; CKCS: Cavalier King Charles spaniel; CFPP: Caudal fossa parenchyma percentage; MC: Mesaticephalic dog; MRI: Magnetic resonance imaging; MTCV: Metencephalon volume; SM: Syringomyelia; TE: Time of echo; TR: Time of repetition.

## Competing interests

None of the authors has a financial or personal relationship with other people or organisations that could inappropriately influence or bias the content of the paper.

## Authors’ contributions

MS and MK determined the volumes of the brains. NO and KA participated in the design of the study and KF performed the statistical analysis. MS, MK and NO conceived of the study, and participated in its design and coordination and helped to draft the manuscript. All authors read and approved the final manuscript
